# Effect of different oral anticoagulants on cognitive function in patients with atrial fibrillation: A Bayesian network meta-analysis

**DOI:** 10.1097/MD.0000000000037750

**Published:** 2024-04-26

**Authors:** Wanling Ning, Shiheng Wang, Hanqing Tang, Sichu Wu, XiaoSong Huang, Baiyan Liu, Yilin Mao

**Affiliations:** a Hunan University of Chinese Medicine, Changsha, China; b China Institute for History of Medicine and Medical Literature, China Academy of Chinese Medical Sciences, Beijing, China; c School of Basic Medicine, Youjiang Medical University for Nationalities, Baise, China; d Changhai Hospital of Shanghai, Shanghai, China; e Hunan Provincial Brain Hospital, Changsha, China; f Hunan Academy of Chinese Medicine, Changsha, China; g The Second Affiliated Hospital of Hunan University of Chinese Medicine, Changsha, China.

**Keywords:** atrial fibrillation, Bayesian network meta-analysis, cognitive function, correlation, oral anticoagulants, system evaluation

## Abstract

**Background:**

Atrial fibrillation (AF) is 1 of the most common types of arrhythmias. At present, the treatment for patients with AF mainly includes oral anticoagulants (OACs). Studies have shown that OACs are associated with cognitive decline in patients with atrial fibrillation; however, there is a lack of relevant evidence. This study used Bayesian network meta-analysis (NMA) to investigate the effects of different oral anticoagulants on cognitive decline in patients with AF.

**Methods:**

We systematically searched for clinical studies on oral anticoagulants in patients with AF in PubMed, Web of Science, Embase, and the Cochrane Library as of July 3, 2023. Cochrane’s randomized controlled trial bias risk assessment tool and the Newcastle–Ottawa Scale were used to assess the bias risk of the included studies. The main outcome measure was decreased cognitive functioning.

**Results:**

Ten studies were included, including 2 RCTs and 7 RCSs, including 882,847 patients with AF. Five oral anticoagulants and 2 anticoagulants were included: VKAs (especially warfarin), Dabigatran, Edoxaban, Rivaroxaban, Apixaban, and Aspirin, Clopidogrel. The results of the mesh meta-analysis showed that VKAs were superior to warfarin in reducing the risk of cognitive decline in patients with AF (OR = −1.19, 95% CI (−2.35, −0.06), *P* < .05) (Table 5). The top 3 drugs in terms of the probability of reducing the incidence of cognitive impairment in patients with AF with different oral anticoagulants were VKAs (87%), rivaroxaban (62.2%), and dabigatran (60.8%).

**Conclusion:**

Based on the results of this study, VKAs may be the best intervention measure for reducing the risk of cognitive decline in patients with AF. Owing to the limitations of this study, more high-quality randomized controlled trials with large sample sizes and multiple centers are required to provide more evidence.

## 1. Introduction

Atrial fibrillation (AF) is 1 of the most common types of arrhythmias. This refers to the sudden occurrence of abnormal electrical pulses in the atrium that exceed the regulation of the natural pacemaker in the heart. AF occurs when the natural pacemaker of the heart is no longer able to control its rhythm. At this point, regular and orderly atrial electrical activity is lost, leading to the occurrence of irregular fibrillation waves. This condition is a serious disorder of atrial electrical activity. AF can cause irregular and usually abnormally rapid contraction of atrial myocardial cells, leading to various symptoms including irregular heart rate, palpitations, dizziness, shortness of breath, and fatigue.^[1]^ AF mainly occurs in the elderly (>70 yrs old) and in people with lifestyle-related diseases such as hypertension, diabetes, and obesity.^[2]^ With the increase in the aging population, the incidence rate of AF is also rising. The incidence rate of AF is estimated to increase by more than 60% by 2050.^[3]^ AF may seriously affect the quality of life of patients because it may lead to serious complications such as stroke, heart failure, cognitive decline, and cardiac arrest, which will lead to an increase in the incidence rate and mortality.^[4]^ A systematic review suggested that AF increases the risk of cognitive impairment, all-cause dementia, vascular dementia, and Alzheimer’s disease (AD) (RR = 2.2, 95% CI = 1.4–3.5, *P* < .05).

At present, the treatment for patients with AF mainly includes controlling the heart rate, converting to sinus rhythm, and anticoagulant therapy, among which permanent AF patients require long-term anticoagulant therapy.^[5,6]^ Antithrombotic drugs, especially oral anticoagulants are the main treatment for most patients with AF. Vitamin K antagonists (VKAs), and direct oral anticoagulants (NOAC), such as dabigatran, rivaroxaban, and apixaban, are widely used in the treatment of AF with good results.^[7]^ Aspirin and clopidogrel are anti aggregation drugs, which are widely used in clinical anticoagulation therapy and therefore included in the study for comparison. Research has shown that abnormal hemostasis can identify the risk of dementia, whereas anticoagulation can prevent the occurrence of dementia.^[8]^ Multiple systematic reviews have shown that OACs can significantly reduce the risk of cognitive impairment. For example, Zong Yuan Lee’s study showed that NOAC had a better effect on reducing dementia than warfarin (OR = 0.65, 95% CI = 0.34, 1.25, *P* < .05).^[9]^ Pajaree Mongkhon research has shown that OAC can reduce the risk of dementia (RR = 0.79, 95% CI = 0.67–0.93, *P* < .05).^[10]^ Cheng et al found that NOAC can reduce the risk of cognitive impairment compared to warfarin (HR = 0.51, 95% CI = 0.37–0.71, *P* < .05).^[11]^ However, the effectiveness of different anticoagulants in reducing cognitive impairment remains unclear.

NMA is a state-of-the-art evidence-based technique that uses direct or indirect comparisons to compare the effects of multiple interventions on diseases and estimate the hierarchical order of each treatment.^[12]^ Therefore, in this study, we used a NMA to compare the cognitive outcomes of different oral anticoagulants (warfarin, dabigatran, rivaroxaban, and apixaban) and aspirin, in patients with AF and provide evidence-based recommendations for patients and clinical physicians.

## 2. Materials and methods

### 2.1. Registration

This study was conducted according to the reporting guidelines of system review and mesh meta-analysis (PRISMA-NMA) and has been conducted in PROSPERO (https://www.crd.york.ac.uk/PROSPERO/#myprospero) Conduct prospective registration (ID: CRD42023420169).

### 2.2. Retrieval strategy

Research papers were retrieved from PubMed, EMbase, Cochrane Library, Web of Science, CNKI, Wanfang, VIP database, and SinoMed. The search encompassed the period from database inception to July 3, 2022. Retrieval strategies were systematically constructed using the PICOS framework as follows: study participants: patients with AF; intervention measures: oral anticoagulants; control group: oral anticoagulants (different from the intervention group); outcome indicators: cognitive impairment; study type: clinical research. Table 1 shows the retrieval strategy used in this study (PubMed).

**Table 1:** Search strategy on PubMed.

#1	Anticoagulants[MeSH Terms]
#2	(((((((Anticoagulants[Title/Abstract]) OR (anticoagulant[Title/Abstract])) OR (Anticoagulation[Title/Abstract])) OR (Indirect Thrombin Inhibitors[Title/Abstract])) OR (anticoagulating agent[Title/Abstract])) OR (anticoagulative agent[Title/Abstract])) OR (Antithrombotic[Title/Abstract])
#3	#1 OR #2
#4	((((((((Warfarin[MeSH Terms]) OR (Dabigatran[MeSH Terms])) OR (Rivaroxaban[MeSH Terms])) OR (apixaban[MeSH Terms])) OR (edoxaban[MeSH Terms])) OR (betrixaban[MeSH Terms])) OR (Aspirin[MeSH Terms])) OR (Clopidogrel[MeSH Terms])) OR (Receptors, Purinergic P2Y12[MeSH Terms])
#5	(((((((((((((((((((((((((((((((((((((((((((((((((Warfarin[Title/Abstract]) OR (4-Hydroxy-3-(3-oxo-1-phenylbutyl)-2H-1-benzopyran-2-one[Title/Abstract])) OR (Aldocumar[Title/Abstract])) OR (Coumadin[Title/Abstract])) OR (Marevan[Title/Abstract])) OR (Coumadine[Title/Abstract])) OR (Dabigatran[Title/Abstract])) OR (Pradaxa[Title/Abstract])) OR (Rivaroxaban[Title/Abstract])) OR (Xarelto[Title/Abstract])) OR (apixaban[Title/Abstract])) OR (Eliquis[Title/Abstract])) OR (aboxoma[Title/Abstract])) OR (eliques[Title/Abstract])) OR (lunast[Title/Abstract])) OR (edoxaban[Title/Abstract])) OR (Savaysa[Title/Abstract])) OR (lixiana[Title/Abstract])) OR (roteas[Title/Abstract])) OR (betrixaban[Title/Abstract])) OR (BEVYXXA[Title/Abstract])) OR (Aspirin[Title/Abstract])) OR (acetylsalicylic acid[Title/Abstract])) OR (2-(Acetyloxy)benzoic Acid[Title/Abstract])) OR (Acylpyrin[Title/Abstract])) OR (Aloxiprimum[Title/Abstract])) OR (Colfarit[Title/Abstract])) OR (Dispril[Title/Abstract])) OR (Easprin[Title/Abstract])) OR (Ecotrin[Title/Abstract])) OR (Endosprin[Title/Abstract])) OR (Magnecyl[Title/Abstract])) OR (Micristin[Title/Abstract])) OR (Polopirin[Title/Abstract])) OR (Polopiryna[Title/Abstract])) OR (Solprin[Title/Abstract])) OR (Solupsan[Title/Abstract])) OR (Zorprin[Title/Abstract])) OR (Acetysal[Title/Abstract])) OR (Clopidogrel[Title/Abstract])) OR (Iscover[Title/Abstract])) OR (Plavix[Title/Abstract])) OR (Receptors, Purinergic P2Y12[Title/Abstract])) OR (Purinergic P2Y12 Receptors[Title/Abstract])) OR (Purinoceptor P2Y12[Title/Abstract])) OR (P2Y12 Purinoceptors[Title/Abstract])) OR (P2Y(ADP) Receptor[Title/Abstract])) OR (P2Y(T) Receptor[Title/Abstract])) OR (P2Y12 Purinoceptor[Title/Abstract])) OR (Purinoceptor P2T[Title/Abstract])
#6	#4 OR #5
#7	#3 OR #6
#8	Atrial Fibrillation[MeSH Terms]
#9	(((((Atrial Fibrillation[Title/Abstract]) OR (Atrial Fibrillations[Title/Abstract])) OR (Auricular Fibrillation[Title/Abstract])) OR (Auricular Fibrillation [Title/Abstract])) OR (atrium fibrillation[Title/Abstract])) OR (heart fibrillation atrium[Title/Abstract])
#10	#8 OR #9
#11	((Cognition) OR (Cognitions)) OR (Cognitive)
#12	#7 AND #10 AND #11

### 2.3. Inclusion criteria

Study participants: patients diagnosed with AF. There were no limits to race, age, sex, or disease course. Intervention measures: OACs: dabigatran, apixaban, edoxaban, betrixaban, VKAs, receivers, purinergic P2Y12, Avastin, aspirin, clopidogrel. Control group: OACs: dabigatran, apixaban, edoxaban, betrixaban; VKAs, receivers, purinergic P2Y12, Avastin, aspirin, clopidogrel (different from the intervention group). Outcome indicators: cognitive impairment (Mini-mental state examination score, incidence rate of dementia). Research type: Clinical research.

### 2.4. Exclusion criteria

Studies with incomplete data; Nonclinical studies, such as reviews, abstracts, letters, and plans. Research on intervention measures that do not meet the inclusion criteria.

### 2.5. Literature screening

Automatic plagiarism-checking function of the Endnote literature management software was used to screen the literature. Two researchers (Ning WL and Wang SH) initially identified duplicate literature. Subsequently, they screened the remaining articles by reading the title and abstract to determine inclusion and exclusion. Finally, the entire text was read to further confirm inclusion. Two researchers independently screened and compared the remaining studies. If they were the same, they were included in the study. If they differed, they were discussed and resolved by a third researcher (Tang HQ).

### 2.6. Data extraction

Two researchers (Ning and Wang) designed a data extraction table based on the information needed for the study, including the author, year, study participants, sex, sample size, intervention measures, follow-up time, outcome indicators, quality evaluation information, and other relevant content. When there is inconsistency in the information extracted by 2 people, they should first discuss and resolve it with each other. If this cannot be resolved, the decision should be made through discussion.

### 2.7. Quality evaluation

Two researchers (Ning WL and Wang SH) independently conducted bias risk assessment. Randomized controlled trials were evaluated using the Cochrane Handbook 5.1.0 version of the ROB tool for assessing bias risk in randomized controlled trials.^[13]^ It includes the following 7 fields: random sequence generation; allocation concealment; participant and subject blind methods; result evaluation blind method; completeness of result data; selective reporting; and other biased sources. The experiment was divided into 3 levels according to the number of components that may have a high ROB: high risk (5 or more), moderate risk (3 or 4), and low risk (2 or fewer).

Cohort studies were evaluated using the Newcastle–Ottawa Scale for bias risk assessment.^[14]^ This table represents the score with ★, with a maximum score of 9 ★, and 7 to 9 ★ are relatively high-quality articles. This table was used in this study with several evaluation items assigned to each aspect. When the assigned items meet the requirements, they are represented by ★, with the highest comparability being 2 ★.

### 2.8. Statistical analysis

In the correlation study, all variables were countable, calculated using the odds ratio (OR) and 95% confidence interval (CI). Owing to potential differences between studies, we chose a random-effects model for analysis instead of a fixed-effects model.^[15]^ We used the gem package of R4.2.0 software, and according to the PRISMA-NMA manual,^[16,17]^ Markov chain Monte Carlo simulation chains were used in a Bayesian framework for network meta-analysis (NMA) aggregation and analysis. We used the node method to quantify and prove the consistency between indirect and direct comparisons, calculated through the instructions in the Stata software, if the *P*-value was >.05. A consistency check was conducted.^[18]^

The Stata software was used to present and describe the network diagrams of the different exercise interventions. In the generated network diagram, each node represents a different motor intervention and control condition, and the lines connecting the nodes represent direct positive comparisons between interventions. The size of each node and width of the connecting lines are directly proportional to the number of studies.^[19]^

The efficacy of each indicator was sorted to obtain the surface under the cumulative ranking (SUCRA) and plot the probability ranking in a graph. A percentage was used to represent SUCRA. A larger percentage indicates that the intervention is more effective and a value of zero indicates that the intervention is completely ineffective. Although SUCRA can be effectively rephrased as a percentage of the effectiveness or acceptability of exercise interventions, these scores should be interpreted with caution unless there are actual clinical differences between the interventions.^[20]^

## 3. Results

### 3.1. Literature screening results

**Figure 1.**
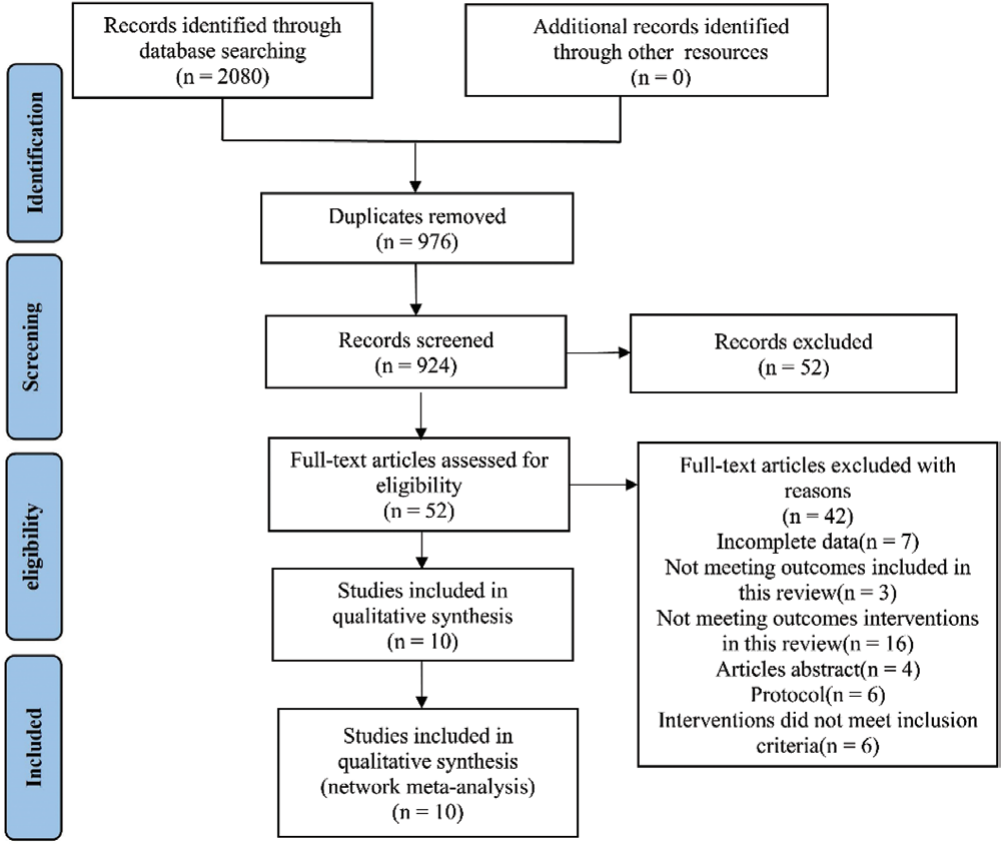
Literature screening process diagram.

In total, 2080 studies were retrieved. A total of 976 duplicate references were excluded: 52 were excluded by reading titles and abstracts, and 42 were excluded by reading the entire text (reasons included incomplete data, noncompliance with the outcome indicators included in this study, conference papers, abstracts, plans, and interventions not included in this study). The remaining ten references were included in this study (Fig. 1).

### 3.2. Basic features

We included a total of 10 studies, including 2 RCTs and 7 RCSs, including 882,847 patients with AF. Five oral anticoagulants and 2 anticoagulants were included: VKAs (especially warfarin), Dabigatran, Edoxaban, Rivaroxaban, Apixaban, and Aspirin, Clopidogrel. The indicators were all indicators of cognitive impairment, with 2 studies using the Monthly Cognitive Assessment (MMSE) and 8 studies calculating the incidence of dementia. Five studies were conducted in the Americas, 1 in East Asia, and 3 in Europe. Patient characteristics are shown in Table 2.

**Table 2:** Basic characteristics of included studies.

Author	Country	Yr	Study type	Population	Age (mean + SD)	Total/male/female	Intervention	Follow-up time	Outcome
M. B A R B E R^[21]^	UK	2004	RCS	AF	70 (66–76), 74 (68–82)	258/119/139	Warfarin/Aspirin	36 months	Dementia
Bruno Caramelli^[22]^	Brazil	2022	RCT	AF	74 (71–77), 76 (72–77)	149/90/59	Dabigatran/Warfarin	12 months	Montreal Cognitive Assessment, MMSE
Nemin Chen^[23]^	USA	2018	RCS	AF	67 (13), 67 (13)	392711/235626/157085	Warfarin/NOAC	72 months	Dementia
Laurie G. Jacobs^[24]^	USA	2009	RCS	AF	82	106/26/80	Warfarin/Aspirin	12 months	Dementia
Victoria Jacobs^[25]^	USA	2016	RCS	AF	73.5 ± 9.6, 71.2 ± 11.9	5254/3100/2154	Warfarin/NOAC	54 months	Dementia
Jiunn-Cherng Lin^[26]^	China	2022	RCS	AF	NA	3514	Aspirin/Clopidogrel/Warfarin	4.86 ± 3.38 year	Dementia
Leif Friberg^[27]^	Sweden	2018	RCS	AF	NA	227646	NOAC/Warfarin	96 months	Dementia
Malini Madhavan^[28]^	USA	2018	RCS	AF	71.9 ± 12.0, 70.4 ± 16.8	2800/1495/1305	Warfarin/VKAs/Aspirin	3 months	Dementia
Maxim Grymonprez^[29]^	Belgium	2023	RCS	AF	75.7 ± 10.1, 70.2 ± 12.0	237012/235771/1241	NOAC/VKAs	72 months	Dementia
Nahal Mavaddat^[30]^	UK	2014	RCT	AF	NA	973	Warfarin/Aspirin	33 months	MMSE

AF = atrial fibrillation, MMSE = mini-mental state exam, NA = not available, NOAC = nonvitamin K antagonists oral antibiotics, RCS = retrospective cohort study, RCT = randomized controlled trial, UK = United Kingdom, USA = The United States of America, VKAs = vitamin K antagonists.

### 3.3. Quality evaluation

The bias risk assessment results for cohort studies showed that 1 study scored 8 points and 7 studies scored 9 points, indicating high literature quality. Bruno Caramelli et al’s study showed insufficient information in Blinding of output assessment (detection bias), while Nahal Mavaddat et al’s study did not provide sufficient information in Random sequence generation (selection bias), allocation consideration (selection bias), and blinding of output assessment (detection bias). See Tables 3 and 4.

**Table 3:** Bias risk assessment results (RCS).

Study	Selection				Comparability	Outcomes			Score
	Is the case definition adequate?	Is the case definition adequate?	Controls selection	Control definition	Comparability of cases and controls based on the design or analysis	Outcome assessment	Was follow-up long enough for outcomes to occur	Adequacy of follow-up of cohorts	
M. B A R B E R	*	*	*	*	**	*	*	*	9
Nemin Chen	*	*	*	*	**	*	*		8
Laurie G. Jacobs	*	*	*	*	**	*	*	*	9
Victoria Jacobs	*	*	*	*	**	*	*	*	9
Jiunn-Cherng Lin	*	*	*	*	**	*	*	*	9
Leif Friberg	*	*	*	*	**	*	*	*	9
Malini Madhavan	*	*	*	*	**	*	*	*	9
Maxim Grymonprez	*	*	*	*	**	*	*	*	9

**Table 4:** Bias risk assessment results (RCT).

Study	Random sequence generation (selection bias)	Allocation concealment (selection bias)	Blinding of participants and personnel (performance bias)	Blinding of outcome assessment (detection bias)	Incomplete outcome data (attrition bias)	Selective reporting (reporting bias)	Other bias
Bruno Caramelli	Low	Low	Low	unclear	low	low	low
BNahal Mavaddat	Unclear	unclear	Low	unclear	low	low	low

### 3.4. Mesh meta-analysis results

**Figure 2.**
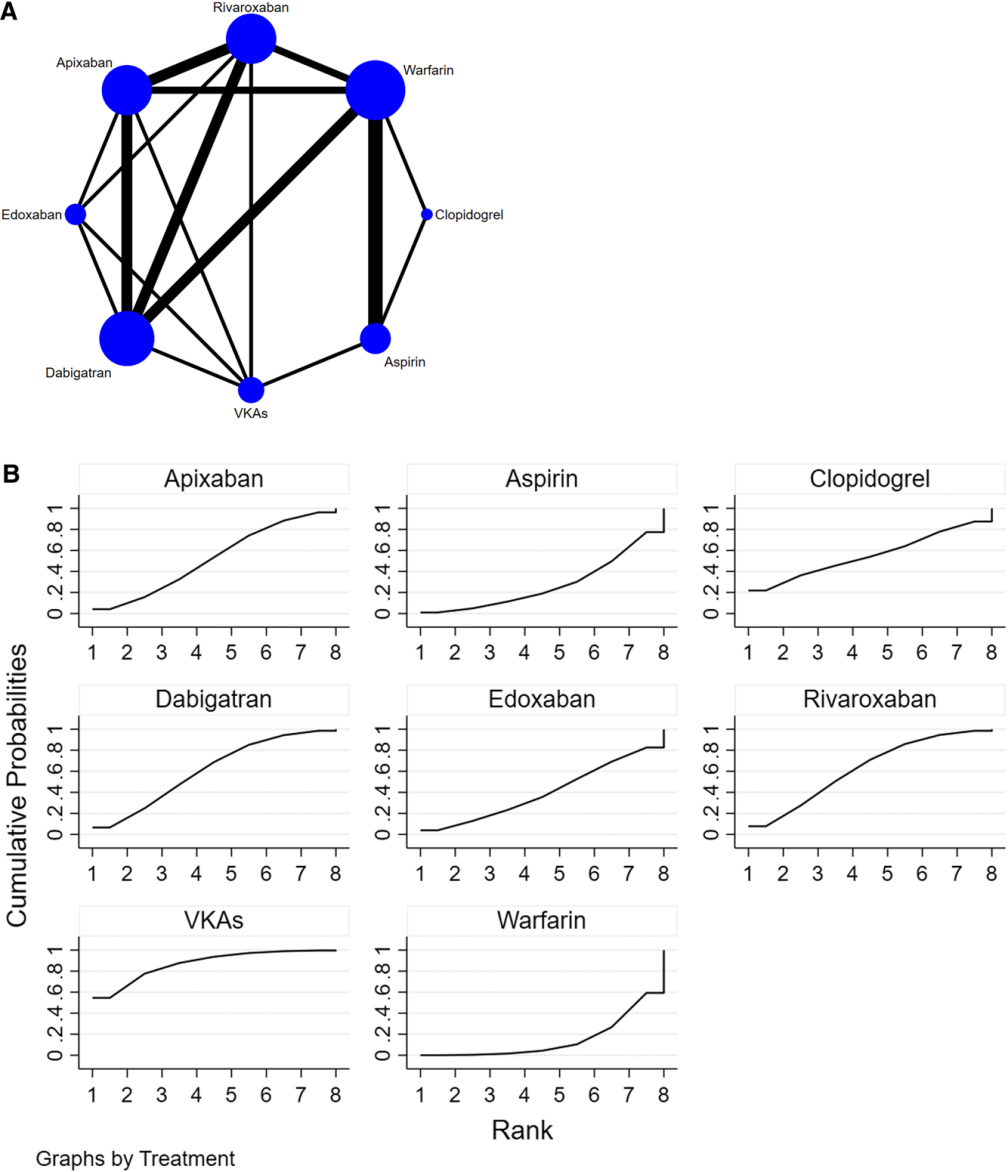
(2A) NMA figure (The blue circle represents the number of users, with larger circles indicating greater participant numbers. Lines represent direct study comparisons; thicker, continuous lines indicate a higher number of studies). (2B) SUCRA plot(The larger the area under the curve, the more effective the intervention measures are). NMA = network meta-analysis.

The complete NMA diagram is shown in Figures 2A and 2B. The network evidence graph showed that there was a closed loop in the study, and the p-values of all indirect and direct comparisons between studies were tested for consistency and inconsistency, with *P*-values >.05, indicating that the consistency effect between studies is acceptable. The results of the mesh meta-analysis showed that other VKAs were superior to warfarin in reducing the risk of cognitive decline in patients with AF (OR = −1.19, 95% CI (−2.35, −0.06), *P* < .05) (Table 5). The top 3 probability rankings of different OACs in reducing the incidence of cognitive impairment in patients with AF were VKAs (87%), rivaroxaban (62.2%), and dabigatran (60.8%), as shown in Figure 2B and Table 6.

**Table 5:**
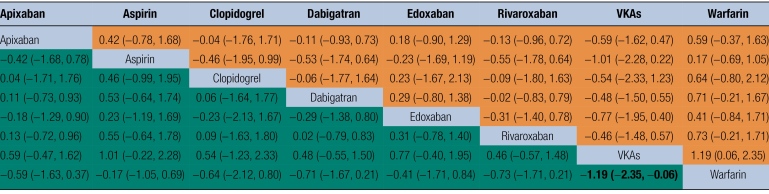
League table.

**Table 6:** SUCRA rank.

Treatment	SUCRA	PrBest	PrBest
Apixaban	52.1%	4.1	4.4
Aspirin	27.7%	1.0	6.1
Clopidogrel	55.3%	21.9	4.1
Dabigatran	60.8%	6.6	3.7
Edoxaban	40%	4.0	5.2
Rivaroxaban	62.2%	7.7	3.6
VKAs	87%	54.5	1.9
Warfarin	14.7%	0.1	7.0

## 4. Discussion

This study included 10 clinical studies on the treatment of AF with OACs, involving 5 OACs and 2 anticoagulants: VKAs(especially warfarin), Dabigatran, Edoxaban, Rivaroxaban, Apixaban, and Aspirin, Clopidogrel. The results of the mesh meta-analysis showed that other VKAs were superior to warfarin in reducing the risk of cognitive decline in patients with AF compared to warfarin. There was no significant difference in the risk of cognitive impairment between the other OACs (*P* > .05). The SUCRA ranking results showed that the VKAs ranked first, indicating that they may be the best OACs and can provide a reference for clinical medication.

VKAs are the most used anticoagulants. Basic research has shown that VKAs may be related to human cognitive function and can act on the central nervous system.^[31]^ Vitamin K participates in brain function by regulating the synthesis of sphingolipids (components of the myelin sheath and neuronal membrane), and in neuronal survival by biologically activating vitamin K-dependent proteins.^[32]^ VKAs can interfere with the vitamin K cycle and reduce the availability of the active form of vitamin K (hydroquinone) in the brain.^[33]^ It regulates the biological activation of 2 types of vitamin K-dependent proteins, namely Gas6 (growth arrest-specific gene 6) and protein S.^[32,34]^ Gas6 participates in chemotaxis, mitosis, cell growth, and differentiation. Amyloid protein-β-induced apoptosis is a hallmark of AD. Protein S protects neurons during ischemia/hypoxia injury.^[35]^ Additionally, vitamin K regulates the metabolism of sphingolipids, which are key participants in neuronal proliferation, differentiation, aging, cell-cell interactions, and transformation.^[32,34]^ Changes in sphingolipid metabolism may also be related to neurodegenerative diseases such as AD. Importantly, the use of VKAs has been shown to alter the distribution of sphingolipids.^[36]^

The connection between VKA use and cognitive impairment has not been fully elucidated. Some clinical studies have demonstrated a correlation between VKAs and cognitive decline. Antoine Brangier collected information on the use of VKAs among 378 elderly outpatient patients. After 12 and 24 months of follow-up, overall cognitive performance and executive function were evaluated using simplified mental state examination and positive assessment group FAB scores, respectively. The results showed that the use of VKAs was associated with lower FAB scores at baseline (adjusted for β=− 2.1, *P* = .026) and correlated with a decrease in FAB scores after 24 months (pre-adjusted β= − 203.6%, *P* = .010). The use of VKAs was not associated with any changes in the mini-mental state examinations scores at baseline (*P* = .655), 12 months (*P* = .603), or 24 months (*P* = .021). Compared with the control group, elderly patients who used VKAs experienced more severe executive dysfunction at baseline and decreased executive power within 24 months.^[37]^ C é drive Anweiler classified 266 elderly patients based on their cognitive impairments. The routine use of VKAs should be examined by asking patients, relatives, and family doctors. Through investigation, it was found that, compared to participants without cognitive impairment, participants with a mini-mental state check of ≤25 used VKAs more frequently (*P* = .038). The use of VKAs was independently associated with cognitive impairment (fully adjusted OR = 17.4 [95% CI: 1.4–224.2], *P* = .028).^[38]^ Guyaine Ferland measured the exposure of VKAs and platelet aggregation inhibitors (another anticoagulant) in 7133 elderly thrombotic patients. Participants underwent cognitive assessment every 2 years at baseline and within 10 years. The results showed a significant correlation between poor performance on the VKAs treatment and the Benton visual retention test (adjusted mean difference, −0.29; *P* = .02 in the multivariate model) for evaluating visual memory at baseline and the Isaacs set test (adjusted mean difference, −1.37; *P* = .0009) to evaluate the language fluency. VKAs treatment was not associated with the overall cognitive function of mini-mental state examinations, nor was it associated with the rate of decrease in scores on all 3 subsequent cognitive tests.^[39]^ The above clinical studies have found a correlation between VKAs and cognitive decline, which may increase the risk of cognitive decline. This is inconsistent with the results of this study, indicating that the correlation between VKAs and cognitive decline is controversial and requires further clinical research.

## 5. Strengths and limitations

Strengths of this study: For the first time, we conducted a NMA of the effects of different OACs on cognitive function in patients with AF, comparing the differences in cognitive function between different OACs in patients with AF. We strictly followed the bias risk assessment method used in clinical research to evaluate the quality of the included studies. We conducted (PRISMA-NMA) according to the reporting guidelines of the system review and mesh meta-analysis and conducted prospective registration with PROSPERO in advance.

Limitations of this study: The number of included studies was relatively small, and there were differences in the selection of efficacy evaluation criteria, patient characteristics, sample size, and outcome indicators in the included studies. These confounding factors may have an impact on the results. The methodological quality of some studies is low, and these factors may have an impact on the strength of the evidence.

## 6. Conclusion

This study compared differences in cognitive function among patients with AF treated with different OACs. Based on the results of this study, VKAs may be the best potential intervention measure to reduce the risk of cognitive decline in patients with AF. Owing to the limitations of this study, more high-quality randomized controlled trials with large samples and multiple centers are needed in the future to provide more evidence.

## Author contributions

**Data curation:** Wanling Ning, Shiheng Wang, Hanqing Tang, Sichu Wu.

**Formal analysis:** Yilin Mao.

**Funding acquisition:** Yilin Mao.

**Investigation:** XiaoSong Huang, Baiyan Liu.

**Resources:** XiaoSong Huang, Baiyan Liu.

**Supervision:** Baiyan Liu.

**Validation:** Wanling Ning, Hanqing Tang, XiaoSong Huang, Yilin Mao.

**Writing – original draft:** Wanling Ning.

## References

[1] Hindricks G Potpara T Dagres N.; ESC Scientific Document Group. 2020 ESC Guidelines for the diagnosis and management of atrial fibrillation developed in collaboration with the European Association for Cardio-Thoracic Surgery (EACTS): the Task Force for the diagnosis and management of atrial fibrillation of the European Society of Cardiology (ESC) Developed with the special contribution of the European Heart Rhythm Association (EHRA) of the ESC. Eur Heart J. 2021;42:373–498. 32860505 10.1093/eurheartj/ehaa612

[2] Brundel BJJM Ai X Hills MT. Atrial fibrillation. Nat Rev Dis Primers. 2022;8:21. 35393446 10.1038/s41572-022-00347-9

[3] Lippi G Sanchis-Gomar F Cervellin G. Global epidemiology of atrialfibrillation: an increasing epidemic and public health challenge. Int J Stroke. 2020;19:1747493019897870. 10.1177/1747493019897870 31955707

[4] Zoni-Berisso M Lercari F Carazza T. Epidemiology of atrial fibrillation: European perspective. Clin Epidemiol. 2014;6:213–20. 24966695 10.2147/CLEP.S47385 PMC4064952

[5] Papanastasiou CA Theochari CA Zareifopoulos N. Atrial fibrillation is associated with cognitive impairment, all-cause dementia, vascular dementia, and alzheimer’s disease: a systematic review and meta-analysis. J Gen Intern Med. 2021;36:3122–35. 34244959 10.1007/s11606-021-06954-8 PMC8481403

[6] Potpara T Dagres N Arbelo E. 2020 ESC Guidelines for the diagnosis and management of atrial fibrillation developed in collaboration with the European Association of Cardio-Thoracic Surgery (EACTS). Eur Heart J. 2021;42:373–498. 32860505 10.1093/eurheartj/ehaa612

[7] Lobraico-Fernandez J Baksh S Nemec E. Elderly bleeding risk of direct oral anticoagulants in nonvalvular atrial fibrillation: a systematic review and meta-analysis of cohort studies. Drugs R D. 2019;19:235–45. 31127504 10.1007/s40268-019-0275-y PMC6738514

[8] Barber M Campbell Tait R Scott J. Dementia in subjects with atrial fibrillation: haemostatic functionand the role of anticoagulation. J Thromb Haemost. 2004;2:1873–8. 15550013 10.1111/j.1538-7836.2004.00993.x

[9] Lee ZX Ang E Lim XT. Association of risk of dementia with direct oral anticoagulants versus warfarin use in patients with non-valvular atrial fibrillation: a systematic review and meta-analysis. J Cardiovasc Pharmacol. 2021;77:22–31. 33136766 10.1097/FJC.0000000000000925

[10] Mongkhon P Naser AY Fanning L. Oral anticoagulants and risk of dementia: a systematic review and meta-analysis of observational studies and randomized controlled trials. Neurosci Biobehav Rev. 2019;96:1–9. 30391408 10.1016/j.neubiorev.2018.10.025

[11] Cheng W Liu W Li B. Relationship of anticoagulant therapy withcognitive impairment among patients with atrial fibrillation: a meta-analysis and systematic review. J Cardiovasc Pharmacol. 2018;71:380–7. 29528873 10.1097/FJC.0000000000000575

[12] Rouse B Chaimani A Li T. Network meta-analysis: an introduction for clinicians. Intern Emerg Med. 2017;12:103–11. 27913917 10.1007/s11739-016-1583-7 PMC5247317

[13] Higgins J Altman D Gotzsche PC. The cochrane collaboration’s tool for assessing risk of bias in randomised trials. BMJ. 2011;343:d5928. 22008217 10.1136/bmj.d5928 PMC3196245

[14] Stang A. Critical evaluation of the Newcastle-Ottawa scale for the assessment of the quality of nonrandomized studies in meta-analyses. Eur J Epidemiol. 2010;25:603–5. 20652370 10.1007/s10654-010-9491-z

[15] Jackson D Riley R White IR. Multivariate meta-analysis: potential and promise. Stat Med. 2011;30:2481–98. 21268052 10.1002/sim.4172 PMC3470931

[16] Moher D Shamseer L Clarke M.; PRISMA-P Group. PRISMA-P group preferred reporting items for systematic review and meta-analysis protocols (PRISMA-P) 2015 statement. Syst Rev. 2015;4:1. 25554246 10.1186/2046-4053-4-1 PMC4320440

[17] Vats D Flegal JM Jones GL. Multivariate output analysis for Markov Chain Monte Carlo. Biometrika. 2019;106:321–37.

[18] Salanti G Ades AE Ioannidis J. Graphical methods and numerical summaries for presenting results from multiple-treatment meta-analysis: an overview and tutorial. J Clin Epidemiol. 2011;64:163–71. 20688472 10.1016/j.jclinepi.2010.03.016

[19] Chaimani A Higgins J Mavridis D. Graphical tools for network meta-analysis in STATA. PLoS One. 2013;8:e76654. 24098547 10.1371/journal.pone.0076654 PMC3789683

[20] Marotta N Demeco A Moggio L. Comparative effectiveness of breathing exercises in patients with chronic obstructive pulmonary disease. Complement Ther Clin Pract. 2020;41:101260. 33221632 10.1016/j.ctcp.2020.101260

[21] Barber M Tait RC Scott J. Dementia in subjects with atrial fibrillation: hemostatic function and the role of anticoagulation. J Thromb Haemost. 2004;2:1873–8. 15550013 10.1111/j.1538-7836.2004.00993.x

[22] Caramelli B Yu PC Cardozo FAM. Effects of dabigatran versus warfarin on 2-year cognitive outcomes in old patients with atrial fibrillation: results from the GIRAF randomized clinical trial. BMC Med. 2022;20:374. 36284318 10.1186/s12916-022-02563-2 PMC9598018

[23] Chen N Lutsey PL MacLehose RF. Association of oral anticoagulant type with risk of dementia among patients with nonvalvular atrial fibrillation. J Am Heart Assoc. 2018;7:e009561. 30571385 10.1161/JAHA.118.009561 PMC6404188

[24] Jacobs LG Billett HH Freeman K. Anticoagulation for stroke prevention in elderly patients with atrial fibrillation, including those with falls and/or early-stage dementia: a single-center, retrospective, observational study. Am J Geriatr Pharmacother. 2009;7:159–66. 19616184 10.1016/j.amjopharm.2009.06.002

[25] Jacobs V May HT Bair TL. Long-term population-based cerebral ischemic event and cognitive outcomes of direct oral anticoagulants compared with Warfarin among long-term anticoagulated patients for atrial fibrillation. Am J Cardiol. 2016;118:210–4. 27236255 10.1016/j.amjcard.2016.04.039

[26] Lin JC Li CH Chen YY. Rhythm control better prevents dementia than rate control strategies in patients with atrial fibrillation-a nationwide cohort study. J Pers Med. 2022;12:572. 35455688 10.3390/jpm12040572 PMC9025212

[27] Friberg L Rosenqvist M. Less dementia with oral anticoagulation in atrial fibrillation. Eur Heart J. 2018;39:453–60. 29077849 10.1093/eurheartj/ehx579

[28] Madhavan M Hu TY Gersh BJ. Efficacy of Warfarin anticoagulation and incident dementia in a community-based cohort of atrial fibrillation. Mayo Clin Proc. 2018;93:145–54. 29329798 10.1016/j.mayocp.2017.09.021 PMC5814135

[29] Grymonprez M Petrovic M De Backer TL. Comparing the risk of dementia in subjects with atrial fibrillation using non-vitamin K antagonist oral anticoagulants versus vitamin K antagonists: a Belgian nationwide cohort study. Age Ageing. 2023;52:afad038. 36934339 10.1093/ageing/afad038 PMC10024890

[30] Mavaddat N Roalfe A Fletcher K. Warfarin versus aspirin for prevention of cognitive decline in atrial fibrillation: randomized controlled trial (Birmingham Atrial Fibrillation Treatment of the Aged Study). Stroke. 2014;45:1381–6. 24692475 10.1161/STROKEAHA.113.004009

[31] Ansell J Hirsh J Hylek E. Pharmacology and management of the vitamin K antagonists: American College of Chest Physicians evidence-based clinical practice guidelines. Chest. 2008;133:160S–98S. 18574265 10.1378/chest.08-0670

[32] Ferland G. Vitamin K and the nervous system: an overview of its actions. Adv Nutr. 2012;3:204–12. 22516728 10.3945/an.111.001784 PMC3648721

[33] Yagami T Ueda K Asakura K. Gas6 rescues cortical neurons from amyloid beta protein-induced apoptosis. Neuropharmacology. 2002;43:1289–96. 12527478 10.1016/s0028-3908(02)00333-7

[34] Ferland G. Vitamin K, an emerging nutrient in brain function. Biofactors. 2012;38:151–7. 22419547 10.1002/biof.1004

[35] Liu D Guo H Griffin JH. Protein S confers neuronal protection during ischemic/hypoxic injury in mice. Circulation. 2003;107:1791–6. 12665496 10.1161/01.CIR.0000058460.34453.5A

[36] Sundaram KS Lev M. Warfarin administration reduces synthesis of sulfatides and other sphingolipids in mouse brain. J Lipid Res. 1988;29:1475–9. 3241123

[37] Brangier A Ferland G Rolland Y. Vitamin K antagonists and cognitive decline in older adults: a 24-month follow-up. Nutrients. 2018;10:666. 29794977 10.3390/nu10060666 PMC6024671

[38] Annweiler C Ferland G Barberger-Gateau P. Vitamin K antagonists and cognitive impairment: results from a cross-sectional pilot study among geriatric patients. J Gerontol A Biol Sci Med Sci. 2015;70:97–101. 25151653 10.1093/gerona/glu133

[39] Ferland G Feart C Presse N. Vitamin K antagonists and cognitive function in older adults: the three-city cohort study. J Gerontol A Biol Sci Med Sci. 2016;71:1356–62. 26576841 10.1093/gerona/glv208 PMC5018559

